# Inhibition of LPS-Induced Oxidative Damages and Potential Anti-Inflammatory Effects of *Phyllanthus emblica* Extract via Down-Regulating NF-κB, COX-2, and iNOS in RAW 264.7 Cells

**DOI:** 10.3390/antiox8080270

**Published:** 2019-08-02

**Authors:** Hui Min-David Wang, Ling Fu, Chia Chi Cheng, Rong Gao, Meng Yi Lin, Hong Lin Su, Nathania Earlene Belinda, Thi Hiep Nguyen, Wen-Hung Lin, Po Chun Lee, Liang Po Hsieh

**Affiliations:** 1 College of Oceanology and Food Science, Quanzhou Normal University, Quanzhou 362000, China; 2 Graduate Institute of Biomedical Engineering, National Chung Hsing University, Taichung City 402; Taiwan; 3 Graduate Institute of Medicine, College of Medicine, Kaohsiung Medical University, Kaohsiung 807, Taiwan; 4 Department of Medical Laboratory Science and Biotechnology, China Medical University, Taichung City 404, Taiwan; 5 Department of Life Science, National Chung Hsing University, Taichung City 402, Taiwan; 6 Deloitte Institute of Biology, Yangtze River Delta Research Institute, Tsinghua University, Beijing 100084, China; 7 Department of Chemical and Materials Engineering, Tunghai University Taichung City 407, Taiwan; 8 Undergraduate Study Program of Biomedical Engineering, Department of Physics, Faculty of Science & Technology, Airlangga University, Surabaya 60115, Indonesia; 9 Tissue Engineering and Regenerative Medicine Laboratory, Department of Biomedical Engineering, International University, Vietnam National University, Ho Chi Minh City 700000, Vietnam; 10 Department of Biomedical Informatics, Postdoctoral researcher for Biomedical Informatics, National Defense Medical Center, Taipei 114, Taiwan; 11 Cardiovascular Clinic, Kaohsiung Armed Forces General Hospital, Kaohsiung City 802, Taiwan; 12 Neurology, Internal Medicine, Cheng Ching Hospital, Taichung City 407, Taiwan

**Keywords:** *Phyllanthus emblica*, antioxidant, COX-2, iNOS, NF-κB

## Abstract

*Phyllanthus emblica* is an edible nutraceutical and functional food in the Asia area with medicinal and nutritive importance. The fruit extract of *P. emblica* is currently considered to be one of the effective functional foods for flesh maintenance and disease treatments because of its antioxidative and immunomodulatory properties. We examined the antioxidant abilities of the fruit extract powder by carrying out 2,2-diphenyl-1-picrylhydrazyl (DPPH) free radical scavenging, iron reducing power, and metal chelating activity analysis and showed excellent antioxidative results. In 3-(4,5-dimethylthiazol-2-yl)-2,5-diphenyltetrazolium bromide (MTT) assay, the result showed that the samples had no cytotoxic effect on RAW 264.7 cells even at a high concentration of 2 mg/mL. To investigate its immunomodulatory function, our estimation was to treat it with lipopolysaccharide (LPS) in RAW 264.7 cells to present anti-inflammatory capacities. The extract decreased reactive oxygen species (ROS) production levels in a dose-dependent manner measured by flow cytometry. We also examined various inflammatory mRNAs and proteins, including nuclear factor-κB (NF-κB), inducible nitric oxide synthases (iNOS), and cyclooxygenase-2 (COX-2). In quantitative reverse transcription polymerase chain reaction (qRT-PCR) and western blotting assay, all three targets were decreased by the extract, also in a dose-dependent manner. In conclusion, *P. emblica* fruit extract powder not only lessened antioxidative stress damages, but also inhibited inflammatory reactions.

## 1. Introduction

Innate immune response, also called nonspecific immune response, is the first barrier to stop detrimental materials invading our bodies and granulocytes, macrophages, and inflammatory biomolecules are involved. Inflammation, a common but complex reaction after the immune system recognizes external pathogens or damaged cells, occurs in all types of human tissues and usually presents a protective effect. Thus, a normal inflammatory response has been regarded as a guard to protect the human body from extrinsic pathogens and intrinsic injury [1]. Vital physiological symptoms, for example, increased blood flow, vasodilation, elevated cellular metabolism, a release of proinflammatory mediators, cellular influx, and an accumulation of fluid are hallmarks of inflammatory responses. Generally, an inflammatory reaction is good for humans. However, abnormal inflammation has been reported to be related to several human chronic diseases, including rheumatoid arthritis, atherosclerosis, and diabetes [2,3]. To heal immoderate inflammation, proinflammatory mediators are aimed as targets because inflammatory cells recruit these materials to the scene site. 

Proinflammatory mediators such as nuclear factor-κB (NF-κB), cyclooxygenase-2 (COX-2) and inducible nitric oxide synthase (iNOS) are pivotal to the evaluation of inflammation levels. Incorrect regulation of NF-κB has been reported to be linked to cancers, inflammatory and autoimmune diseases, viral and bacterial infections, and improper immune responses [4]. Because there is a variety of proinflammatory gene expressions induced by NF-κB and the regulation of inflammation, down-regulating of NF-κB activation contributes to various inflammatory diseases [5]. NF-κB also participates in the transcription of another inflammatory association enzyme, iNOS. Dependent activation of the iNOS promoter supports an inflammation-mediated stimulation of this transcript. Nitric oxide (NO) is a critical signaling molecule as a retrograde neurotransmitter which is associated with neural development, immune response, angiogenesis, and one vital feature of inflammation, i.e., vasodilation [6]. NO is mediated in humans by three major types of nitric oxide synthases (NOS) (i.e., endothelial NOS (eNOS), neuronal NOS (nNOS), and iNOS) [7]. When iNOS is activated by cytokines, NO is released. NO is an activating factor of cyclooxygenase (COX), which forms a five coordination with the COX structure, causing a conformational change in COX. COX is officially called prostaglandin endoperoxide synthase, and it is responsible for the biosynthesis of prostanoid, such as thromboxane and prostaglandins, from arachidonic acid. In humans, one of two cyclooxygenases, COX-2, responds by mediating inflammatory reactions [8]. Therefore, COX-2 inhibitors are often used as anti-inflammatory drugs.

*Phyllanthus emblica* fruit, an Indian traditional medicine and an effective functional food, has been used to test its anti-inflammatory activity for centuries, and provides potential therapeutics for a variety of maladies [9]. *P. emblica* fruit contains high levels of vitamin C, tannins, polyphenols (gallic acid and ellagic acid), minerals, fibers, and so on [10]. Recently, several hydrolysable tannins, flavonoids, and alkaloids have been identified in *P. emblica* fruit. Not surprisingly, vitamin C, gallic acid, and ellagic acid, which are present in *P. emblica* fruit, are known to be potent antioxidants, flavonoids, and other biofunctional constitutes that assist inflammation reduction [11]. Although some materials have been proven to improve the symptoms of the inflammation, the mechanism of *P. emblica* fruit on its anti-inflammation activity is still not well known. As an edible food or food additive, *P. emblica* fruit extract powder can be used as an antioxidant and anti-inflammatory diet, and its fruit may help us to deal with these related diseases.

## 2. Materials and Methods

### 2.1. Materials

The testing sample, *P. emblica* fruit extract powder, was obtained from SHENG GUO Biotech Co., Ltd, Miaoli, Taiwan. Dimethyl sulfoxide (DMSO); lipopolysaccharide (LPS) (*Escherichia coli* 055: B5); vitamin C; 2,2-diphenyl-1-picrylhydrazyl (DPPH); ethylenediaminetetraacetic acid (EDTA); 3-tert-butyl-4-hydroxyanisole (BHA); potassium ferricyanide [K_3_Fe(CN)_6_] trichloroacetic acid, FeCl_3_, FeCl_2_·4H_2_O, and 3-(4,5-dimethylthiazol-2-yl)-2,5-diphenyl tetrazolium bromide (MTT); 2,7-dichlorofluorescein diacetate (DCFDA, D6883); and bicinchoninic acid (BCA) were purchased from Sigma-Aldrich Corp., USA. Dulbecco’s Modified Eagle’s Medium (DMEM), fetal bovine serum (FBS), penicillin, streptomycin, and amphotericin B (PSA) were purchased from GIBCO BRL (Gaithersburg, MD, USA).

### 2.2. P. emblica Fruit Powder Extracts Preparation

The extraction of *P. emblica* fruit was carried out using a custom freeze-drying procedure using a freeze dryer (FD-1, CHIANG DING Technology co., ltd., Taiwan) to make the *P. emblica* fruit at −35 °C for 10–12 h, and then dried at 60 °C ± 5% for 35 h. In order to freeze and dry the water in the fruit of *P. emblica* and make it into a powder, after the fruit was freeze dried, the moisture inside the fruit had to be less than 5%, and this was detected using a moisture analyzer (ML-50, A&D Technology, Inc., Japan). The dried fruits were extracted with 85–95 °C hot water at 5 liters per kilogram of fruit to make a liquid extract with 5% soluble content. The extract was filtered through a 10 microns polypropylene filter bag to remove insoluble materials. After vacuum concentration, the concentration was increased to 10% w/v. Maltodextrin was used as the carrier, which was added at a 1:1 ratio (10% *P. emblica* soluble content, 10% maltodextrin, w/v). The concentrate was frozen at −35 °C followed by freeze drying for 72 h (0–50 h at 0 °C, and then by a temperature ramp for 50–72 h to 45 °C) and pulverized to gain testing samples using a 1HPTable Type Pulverizing Machine (Product ID: RT-34).

### 2.3. Free Radical Scavenging Activity

The DPPH reagent which accepts an electron or hydrogen radical becomes a stable molecule to detect oxidative activities. When DPPH reacts with antioxidant agents, hydrogen is supplied, reducing the amount of DPPH and decreasing its absorbance, the optical density (OD) values at 517 nm [12,13]. Compared to other antioxidants, vitamin C (100 μM) is a great positive control because of its prominent antioxidant capacity. We added 1 μL at different concentrations of *P. emblica* fruit extracts and primary-filtered water to 99 μL DPPH (0.1 mg/mL). The absorbance was measured using the spectrophotometer and the remaining DPPH amount was plotted to determine the initial concentration of DPPH reduced by the antioxidant. Various sample amounts were dissolved in methanol for each well, and the final working volume was 100 μL. The clearance capacity (%) is calculated as follows:

$$\mathrm{Clearance}\;\mathrm{capacity}\;(\%)=\frac{\left(A_{\mathrm{blank}}-A_{\mathrm{sample}}\right)}{A_{\mathrm{blank}}}\times 100\%$$

### 2.4. Ferric Reducing Antioxidant Power (FRAP) Assay

We carried out the reducing power assay analysis to examine the reductive ability of *P. emblica* fruit extract samples. The samples were dissolved in DMSO at a suitable concentration mix of 85 μL, phosphate buffer (0.2 M, pH 4.4) and 20% potassium ferricyanide (2.5 μL). The mixture was kept at 50 °C for 20 min, and then 160 μL of 10% trichloroacetic acid (TCA) was added. Subsequently, the solution was centrifuged at 3000× g for 10 min collecting supernatant (75 μL), and 25 μL FeCl_3_ (2%) was added to the supernatant. After a 10 min reaction, the absorbance of the solution was measured at OD_700_ nm [14,15]. BHA was used as a positive control at 100 μM. A higher absorbance value means a better reduction activity.

### 2.5. Metal Chelating Ability Test

The chelation of ferrous ions in our sample was estimated by our previously published method [13]. Briefly, 10 μL of 2 mM FeCl_2_·4H_2_O was added to 1 μL of various concentrations (0.5–50 mg/mL) of samples, and the reaction was initiated by the addition of 20 μL of 5 mM ferrozine. This assay is based on the complexes of ferrous ions and ferrozine that change color at 562 nm, and the lower absorbance means the better metal chelating activity. EDTA at 100 μM acted as a positive control, and the chelating power activity is calculated by:

$$\mathrm{Metal}\;\mathrm{chelating}\;\mathrm{activity}\;(\%)=\frac{\left(A_{\mathrm{control}}-A_{\mathrm{sample}}\right)}{A_{\mathrm{control}}}\times 100\%$$

### 2.6. Cell Culture and Treatment

Mouse macrophage cell lines, RAW 264.7, were purchased from Bioresource Collection and Research Center (BCRC number: 60001) and cultured in DMEM containing 10% FBS and 1% PSA. Cells were incubated at 37 °C in a humidified incubator with 5% CO_2_ atmosphere [16]. Samples were dissolved in DMSO and then diluted by using DMEM medium (0.25, 0.5, 1, and 2 mg/mL). The cells were pretreated with testing samples for 1 h and stimulated with LPS (5 μg/mL) for 6 h, and untreated cells served as a blank control [17,18].

### 2.7. Cell Viability Assay

RAW 264.7 cell viability was evaluated using MTT colorimetric assay [19,20]. The cells were cultured in DMEM containing 10% FBS and 1% PSA at 37 °C in 5% CO_2_. All of the cells were seeded in 96-well microplates. After seeding the cells for 24 h, samples with concentration ranges from 0.005 to 10 mg/mL were added. After another 24 h, cells were treated with MTT solution (0.5 mg/mL) for 2 h, followed by incubation at 37 °C. After 2 h of MTT treatment, the medium was removed, and 100 μL of DMSO was added in each well to dissolve the purple formazan crystals. The dishes were gently shaken for 20 min in the dark to ensure maximal dissolutions of formazan crystals, and OD values of the supernatant were measured at 595 nm. The cell viability was presented as the percentage of live cells in each well, and was calculated according to the following formula:

$$\mathrm{Cell}\;\mathrm{viability}\;(\%)=\frac{\left(A_{\mathrm{sample}}-A_{\mathrm{blank}}\right)}{\left(A_{\mathrm{control}}-A_{\mathrm{blank}}\right)}\times 100\%$$

### 2.8. Measurement of Intracellular ROS Level

The ROS-sensitive fluorescent dye, DCFDA, was used to determine LPS-upregulated intracellular ROS level in RAW 264.7 cells. DCFDA is generally non-fluorescent, but in the presence of ROS (when this reagent is oxidized) it turns into green fluorescent. For an observation of intracellular ROS product through the oxidation of DCFDA, cells were pretreated with or without *P. emblica* samples (0.5–2.0 mg/mL) for 1 h, and stimulated with LPS (5 μg/mL) for 6 h. Afterward, we rinsed them with warm phosphate-buffered saline (PBS) buffer, and incubated them in PBS containing 20 μM DCFDA at 37 °C, 5% CO_2_ for 30 min. PBS containing DCFDA was removed and replaced with fresh cell medium. The cells were washed at least 3 times with PBS and detached with trypsin/ EDTA. The fluorescence intensity of the cells was analyzed using a Guava® easyCyte Flow Cytometers (Merck KGaA, Darmstadt, Germany) at 485 nm excitation and 530 nm emission for 2,7-dichlorofluorescein (DCF) [21].

### 2.9. Quantitative Reverse Transcription Polymerase Chain Reaction (qRT-PCR)

For the qRT-PCR, a 10 μL reaction contained a 3 mixture of two reverse transcriptases: 10 μL of 2 × AceQ qPCR SYBR Green Master Mix (Vazyme Biotech Co.,Ltd, Nanjing, China) with the hot start Taq polymerase, 0.5 μL of primers, and 0.5 μL (20 ng/mL) of template. The primer sequences are listed in Table 1. The StepOnePlus™ System (Version 2.3) was used for all real-time PCR assays [22]. The reaction activated the AceTaq® DNA polymerase at 95 °C for 5 min. This was then amplified for 40 cycles at 95 °C for 3 s for denaturation, annealing, and acquisition at 60 °C for 40 s. It was finally elongated at 95 °C for 15 s. Fluorescence was measured after the annealing phase. With an Applied Biosystems™ MicroAmp™ (N8010560) Fast Optical 96-Well Reaction Plate, 10 μL of the reaction mix was added, as well as the 96-SYBR-Green assays on the StepOnePlus™ Real Time System [23]. To prepare the assay, all of the reagents were kept either on a cooling block or on ice. The ΔΔCt method was used in calculations. For each sample, three independent qPCR experiments were performed. Each experiment involved three replicates for each gene. An expression of GAPDH was used as an internal control. Duplicate SGPERT reactions were performed on each lysate sample. Using qPCR software and instruments, an ABI 7300 with its threshold determined manually and a LightCycler® 480 with its maximum second derivative method generated the cycle of quantification (Cq) values. Using the same software for both instruments, the melting peaks were also automatically calculated.

### 2.10. Western Blotting

A total of 10^5^ cells were treated with sample groups or the blank vehicle control for one day, respectively. The RAW 264.7 cells were harvested and lysed with the lysis buffer (Thermo Scientific Pierce RIPA Buffer, 1 mM EDTA, 10% glycerol, 1% Nonidet P-40, 2 μM leupeptin, 50 mM Tris-HCl, 137 mM sodium chloride, 50 mM sodium fluoride, 10 mM sodium pyrophosphate, 20 mM β-glycerophosphate, 1 mM phenylmethylsulfonyl fluoride, 0.1 mM sodium orthovanadate, and 2 μg/mL aprotinin; pH 7.5). Afterwards, the lysate was cleaved on ice for 30 min, then centrifuged at 12,000 × g for 30 min, and then placed in an incubator for 30 min. The protein quantitation in the supernatant was measured by a BCA protein assay kit. The amounts of protein were taken in equal quantities and separated by sodium dodecyl sulfate-polyacrylamide gel electrophoresis (SDS–PAGE) on 10% gel, and electrotransferred to a polyvinylidene difluoride (PVDF) nitrocellulose membrane (PALL Life Science, Ann Arbor, MI, USA). The transfer film was gently removed from the wet transfer tank, then closed the PVDF membrane with 5% skim milk for 1 h. After this, a mild rinse of 1 × TBS-T was carried out to eliminate any traces of skim milk. In each case, the membrane was incubated with a corresponding anti-mouse primary antibody. In each case, the membrane was incubated with a corresponding anti-mouse and anti-rabbit primary antibody. We used antibodies, including anti-β-actin (St John’s Laboratory, STJ97040), anti-NF-κB (Cell signaling, C22B4), anti-COX-2 (Elabscience, E-AB-27666), and anti-iNOS (Thermo Fisher Corp., PA1-036). We added descriptions of the multiple dilutions of the various primary antibodies as follows: anti-β-actin 1:5000, anti-NF-κB 1:1000, anti-COX-2 1:1000, and anti-iNOS 1:500. Washed at least three times with TBST buffer (TBS containing 0.1% Tween 20) and dipped in horseradish peroxidase-conjugated secondary antibodies against the corresponding primary antibody. Then treated with enhanced chemiluminescence (ECL) detection reagents (PerkinElmer, ECL1:ECL2 = 1:1) and exposed to a Mini Size Chemiluminescent Imaging System from Life Science to specify the time intervals for detecting the protein bands and visualizing the stained blots [24].

### 2.11. Statistical Analysis

All the experiments in each platform were carried out in triplicate and presented as mean ± standard error. For statistical analysis, all data were analyzed by Student’s *t*-test for multiple comparisons. A significant difference (*) was defined as *p* < 0.05.

## 3. Results

### 3.1. Antioxidant Activity of P. emblica Fruit Extracts Powder

As a functional food, antioxidant properties of *P. emblica* samples were assessed using various biochemical assays with different objectives, namely, DPPH, power reducing, and metal chelating activity. The first oxidation inhibitory assay was the DPPH radical scavenging test. This is a simple and economical experimental platform, in which antioxidants act to prevent oxidation products. Antioxidants change the color of the stable radical DPPH reagent from purple to the light yellow of diphenyl-picrylhydrazine. As shown in Table 2, *P. emblica* exhibited excellent radical scavenging ability and scavenged 88.7 ± 0.3% of the DPPH free radical, and vitamin C scavenged 89.9 ± 0.17%. In the power reducing assay, the color of the testing solutions changed from yellow to different shades of green and blue depending upon the reducing power of these antioxidants. The presence of antioxidant substance induces the reduction of the Fe^3+^/ferricyanide complex to the ferrous form. As shown in Table 2, BHA at 100 μM has a reducing power value of 0.6 ± 0.002%, and *P. emblica* at 50 mg/mL has a reducing power value of 2.31 ± 0.05% as compared with BHA. The ferrous ion-chelating activities of *P. emblica* samples are shown in Table 2, and ferrozine could form complexes with Fe^2+^ quantitatively. With the presence of chelating agents, the complex construction was disrupted, resulting in a lightening of the red color of the complex. Compared with EDTA, although the testing samples showed a lower level of Fe^2+^ scavenging ability, its antioxidant activity still showed an increasing trend. *P. emblica* at the concentration of 50 mg/mL presented 16.9% ± 0.11% inhibition. The positive control, EDTA, had approximately 94.4 ± 0.21% ion-chelating capacities at 100 μM.

### 3.2. Cell Viability Effect of P. emblica Fruit Extract Treatment

**Figure 1:**
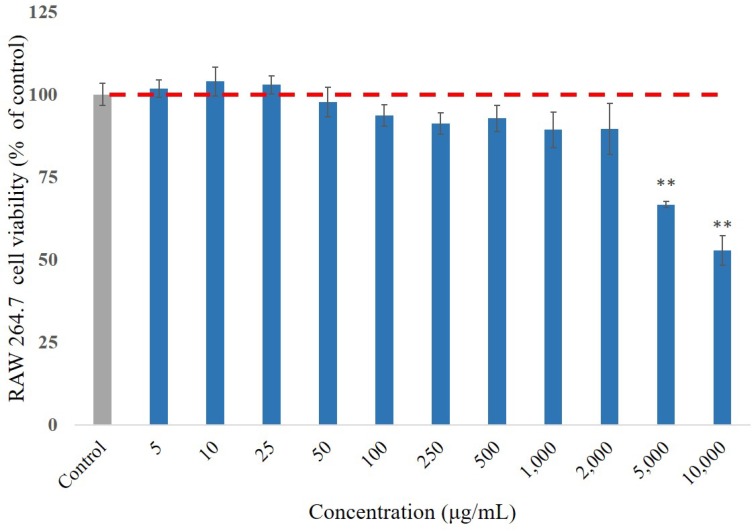
The cell viability of RAW 264.7 cells was measured by MTT assay after 24 h treatment of *P. emblica* samples. The data represented mean ± S.D. of three independent experiments performed. The red dash line is the trend line for cell survival rate of 100%. * *p* < 0.05, ** *p* < 0.01.

As a potent food additive, the component should be harmless, without undesirable cytotoxic side effects. To evaluate the optimal dose of *P. emblica* fruit extract samples, the cytotoxicity of its varying concentrations (0.005–10 mg/mL) were applied to RAW 264.7 cells for 24 h. It was initially determined using MTT assay (Figure 1). The results showed that the low concentrations of the testing samples contributed to proliferations on the RAW 264.7 cells, and had cellular survival rates of 66.7 ± 0.9% and 52.7 ± 2.6% even at high concentrations of 5 and 10 mg/mL, respectively. It proved that the extract of *P. emblica* fruit did not affect the cell viability in RAW 264.7 cells. At 2 mg/mL, the samples had no severe cytotoxic effect on the RAW 264.7 cells, and thus the dosage was optimally deliberated in all the following experiments.

### 3.3. ROS Scavenge by P. emblica Fruit Extract Powder

**Figure 2:**
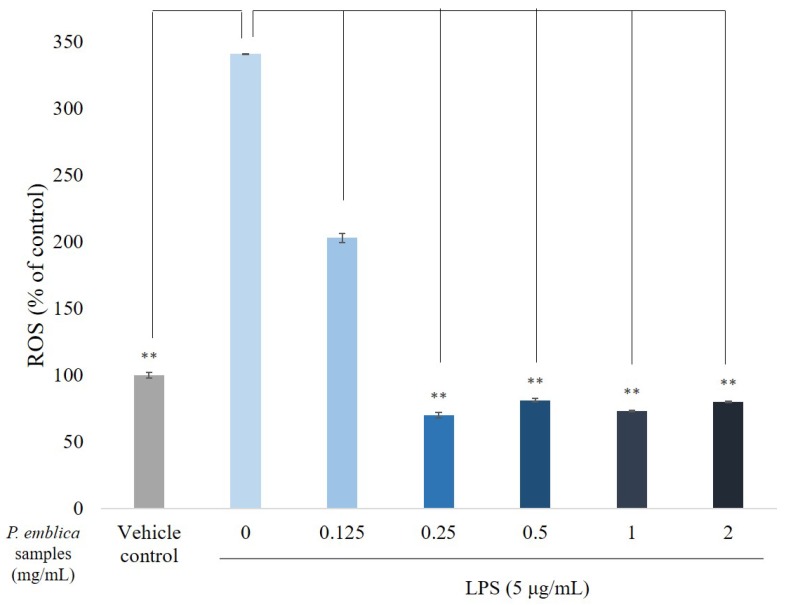
The reactive oxygen species (ROS) percentage measured by a flow cytometry. RAW 264.7 cells were pretreated with *P. emblica* samples (0.125–2 mg/mL) for 1 h, and then stimulated with LPS (5 μg/mL) for 6 h. The data represented mean ± S.D of three independent experiments performed. * *p* < 0.05, ** *p* < 0.01.

To determine whether *P. emblica* fruit extract powder treatment induces cellular oxidative statuses, we investigated ROS generation in RAW 264.7 cells. The intracellular H_2_O_2_ results of the DCFDA staining, which is often quantified to measure the oxidative stress, can be defined as the presence of oxidation. Typically, DCFDA is introduced into target cells through a small amount of aqueous solution, and then rapidly diffuses through the cell membrane as a colorless probe. Once the two acetate groups are cleaved by esterases within the cell, the DCFDA fluorescence is detectable. A valuable property of DCFDA is that it cannot be exited within the cell once it has been cleaved in the cell. This increases the period of time, and DCFDA can be used as a cellular indicator. As shown in Figure 2, increases of *P. emblica* sample concentrations gradually decreased oxidative stresses. DCF fluorescent intensity was reduced to 69.8 ± 0.5% at 0.25 mg/mL, indicating that the treatment of the samples reduced the production of cellular ROS.

### 3.4. Quantitative Reverse Transcription Polymerase Chain Reaction Analysis for NF-κB, iNOS, and COX-2

**Figure 3:**
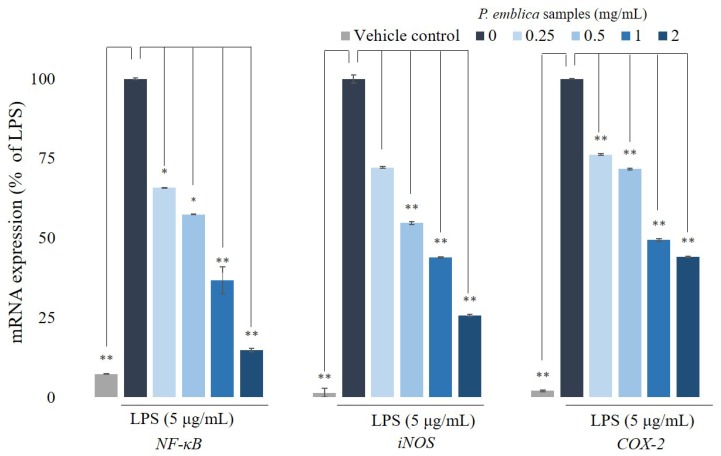
The inflammation-related mRNA expressions in RAW 264.7 cells. RNA expression levels of *NF-κB, iNOS, COX-2* in RAW 264.7 cells treated with different concentrations of *P. emblica* samples (0.25–2 mg/mL) were evaluated by qRT-PCR and normalized to the *GAPDH* gene. The data represented mean ± S.D of three independent experiments performed. * *p* < 0.05, ** *p* < 0.01.

To observe the effect of *P. emblica* fruit extract powder on cytokine expression in RAW 264.7 cells, the cells were pretreated with proper concentrations (0.25–2 mg/mL) for 1 h and then stimulated with LPS (5 μg/mL) for 6 h. When the cells are traumatized or infected by gram-negative bacteria, the bacterial cell wall component, LPS, induces the activation of *NF-κB* triggering inflammatory cytokines. During an inflammation, LPS primarily actuates the reaction of proinflammatory genes, including *iNOS* and *COX-2*, producing significant amounts of NO. The inflammatory mediator gene, *NF-κB*, also plays an important role in inflammation-related diseases, which is related to the above gene modulation expressions. The expressions of *iNOS* and *COX-2* lead to an increased production of proinflammatory bio-molecules, which eventually lead to the progression of inflammatory cytokines. Transcriptional changes in *NF-κB*, *COX-2*, and *iNOS* were confirmed by qRT-PCR, as shown in Figure 3. When cells were stimulated with LPS for 6 h, gene expressions of *NF-κB*, *COX-2*, and *iNOS* were increased. After different concentrations of the extract were incubated with LPS, we observed that the levels of *NF-κB*, *COX-,2* and *iNOS* were reduced to 14.8 ± 0.6%, 25.6 ± 0.4%, and 44.1 ± 0.1%, respectively. 

### 3.5. Western Blotting Analyses for NF-κB, iNOS, and COX-2

**Figure 4:**
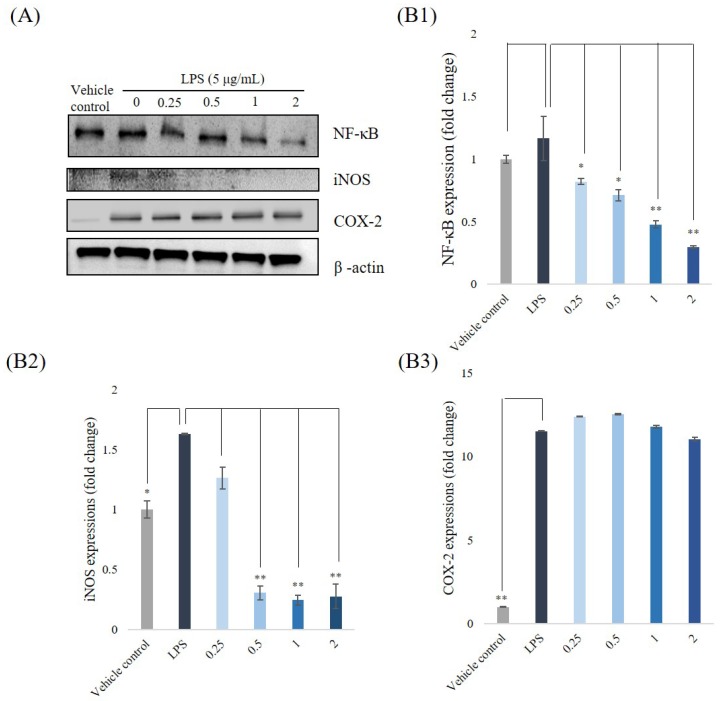
The inflammation-related protein expressions. (**A**) NF-κB, iNOS, and COX-2 expressions in RAW 264.7 cells were pretreated with *P. emblica* samples (0.25–2 mg/mL) for 1 h, and then were stimulated with LPS (5 μg/mL) for 6 h. (**B1**) Protein quantification of NF-κB (**B2**) iNOS (**B3**) COX-2 in western blotting. β-Actin was viewed as an internal control. * *p* < 0.05, ** *p* < 0.01.

We carried out western blotting to analyze the inhibitory effects of *P. emblica* fruit extract powder on expressions of NF-κB, iNOS, and COX-2. The RAW 264.7 cells were treated at fitting sample concentrations, and then stimulated with LPS (5 μg/mL) for 6 h. The inflammatory mediators, NF-κB, iNOS, and COX-2, reflect the states of inflammations and are often used to estimate the severities of the inflammation. The stimulations with LPS led the expressions of three proteins upregulating, as shown in Figure 4A. As we predicted, their levels were down-regulated by *P. emblica* fruit extract to 1.16 ± 0.2%, 1.74 ± 0.06%, and 1.51 ± 0.03%, respectively. Quantifications of the western blotting are shown in Figure 4B1–B3. These results suggest that the extract plays an anti-inflammatory role in LPS-stimulated macrophage RAW 264.7 cells.

## 4. Discussion

Flavonoids naturally have excellent antioxidant capacity, and tannins are known for their anti-inflammation and antioxidant activities. According to one study, several tannins are considered to be potential cytotoxic and anti-inflammatory agents [25]. *P. emblica* fruit in nature is an edible that contains flavonoids, tannins, and other compounds which have excellent antioxidative capacities. Therefore, it can be used in general food for ingestion and as a supplementary food to enhance human health [8]. In view of this, we are interested in the role of *P. emblica* fruit due to the existence of all the above-mentioned important classes of bioactive properties. The main components of the fruit of *P. emblica* include phenolic constituents, flavonoids, polysaccharides, sterols, fatty acids, vitamins, proteins, amino acids, trace elements, anthraquinone, and alkaloids, etc [10]. These main ingredients all have antioxidative potentials. It can be explained that *P. emblica* extract repairs the LPS-induced oxidative damage and inflammatory symptom of RAW 264.7 cells [13].

Free radicals are substances produced by the metabolism of oxygen in the body. They are extremely active and can react strongly with any substance. In physiological conditions, when bacteria, mold, viruses, etc., invade the body, the defense system will notify the immune cells to prepare for the battle in the body [19]. Thus, the immune cells are catalyzed by the enzymes to produce superoxide anion radicals to remove bacteria and infected cells. In other words, the body needs some free radicals as a weapon to prevent disease. Once the free radicals in the body exceeds the normal range, a free radical chain reaction will occur, which will promote the oxidation of proteins, carbohydrates, lipids and other basic constituent substances into new free radicals. In the continuous circulation, the functions of the human body will be corrupted. Antioxidants are chemicals that do not only reduce the rate of oxidation of cells and biomolecules, but also protect the body from free radicals. Adding antioxidant-rich foods can prevent free radical damage. In order to maintain a healthy body we should not only eat a variety of fruits and vegetables in a balanced manner, but also supplementary antioxidants-rich foods [19]. Antioxidant studies showed that *P. emblica* fruit extract has the capacity to either inhibiting free radical ability or to be a free radical scavenger. In this study, we analyzed the DPPH, metal chelating activity, reducing power, and cellular ROS to estimate the free radical scavenging ability of the extract in various concentrations and also carried out qRT-PCR experiments on different inflammatory genes. We confirmed that *P. emblica* fruit extract powder is an effective antioxidant which also has the ability to regulate inflammatory genes.

Once a human gets damaged by foreign objects, the body produces a protective response which is the inflammation. A controlled inflammatory response is beneficial to the body and provides protection against the site of infection. However, once the inflammatory response is dysregulated, it may become harmful. Therefore, the inflammation may evolve into an adaptive response to restore homeostasis. In order to resolve the inflammatory response in the body, the main site of infection promotes the aggregation and mediated of macrophages and T cells which repairs the inflamed parts. The inflammatory response consists of a many media that form a complex regulatory network [26]. Chronic inflammation is also associated with many death-related diseases. Various interconnecting signaling pathways are related to the development of inflammation.

**Figure 5:**
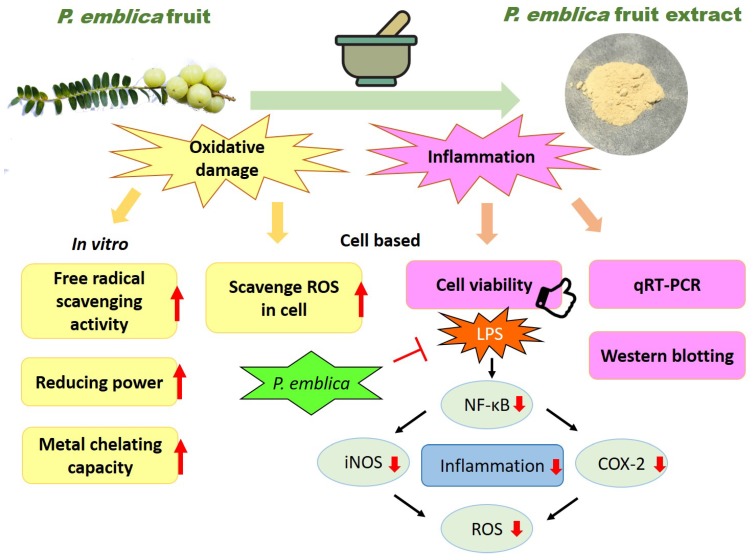
The cartoon illustration of antioxidation and anti-inflammation mechanisms of *P. emblica* fruit extract powder.

NF-κB is an extremely important molecule in the inflammatory reaction. When the cells receive stimulation from the outside of the cell, NF-κB in the cytoplasm is released and activated by the original IκB inhibition. NF-κB is also involved in the transcription of iNOS. When iNOS is activated by cytokines, NO will be released, and NO is an activator of COX [7,8]. Almost all mammal cell types have NF-κB, which consists of a family of transcription factors and is associated with inflammatory cytokine production, cell survival, activation, and differentiation of innate immune cells and inflammatory T helper cells. It regulates a large array of genes which takes part in the immune and inflammatory responses [27]. *NF-κB* is involved in several cellular responses to stimuli such as stress, free radicals, heavy metals, ultraviolet irradiation, oxidized LDL, and pathogens like bacterial or viral antigens. iNOS produces multitudinous amounts of NO that can activate immune cells in inflamed tissue, and thus speed up pathological changes [28]. The proinflammatory cytokines, prostaglandins, and NO are produced by activated macrophages which play decisive roles in inflammatory diseases such as Parkinson’s disease and Alzheimer’s disease. Compared to the critical calcium-calmodulin dependent regulation isoenzymes (nNOS and eNOS), iNOS has been reported as calcium insensitive, maybe due to its tight noncovalent interaction with the calcium-calmodulin complex. iNOS produces larger quantities of NO than eNOS and nNOS upon stimulation, such as by proinflammatory cytokines. iNOS binds calmodulin at physiologically relevant concentrations to synthesize a free radical with an unpaired electron to present an immune defense mechanism. The high iNOS activity typically occurs in an oxidative environmental stimulation, and the overexpressive levels of NO by proinflammatory cytokines have the opportunity to interact with superoxide leading to cell toxicity and peroxynitrite production [27]. These properties may define the roles of iNOS in human immune response, especially the stimulation of inflammation caused by macrophages [8]. Related to the generation of prostaglandin, the major effect which COX-2 causes in inflammation is the generation of pain. Prostaglandin controls the role of vasodilation and inhibits the aggregation of blood plates. In inflammation, these roles have an influence on the accession of blood flow, such as regulating the contraction of smooth muscle tissue and preventing needless clot formation. Thus, COX-2 indirectly increases the sensitization of peripheral nociceptors and generation of hypersensitivity pain. In pathology, several pharmaceutical inhibitions of COX have been used so that they can provide relief caused by the symptoms of inflammation and pain, such as aspirin and ibuprofen [29]. Therefore, the inhibition of proinflammatory cytokines or iNOS and COX-2 expressions in inflammatory cells provides a novel therapeutic method for treatment of inflammation. We used LPS to irritate macrophages as an in vitro model of inflammation. The *P. emblica* sample treatments extenuated LPS-induced inflammation. This study illustrated that iNOS, COX-2, and NF-κB levels increase significantly in LPS-induced cells, whereas, they were evidently decreased by treatment with *P. emblica* fruit extract powder. It means that *P. emblica* fruit samples protect the cell and prevent inflammation symptoms via decreasing the expressions of iNOS, COX-2, and NF-κB at the transcriptional levels and protein expressions, as shown in Figure 5.

## 5. Conclusions

This study is about antioxidative properties and anti-inflammatory effects from *P. emblica* fruit extracts induced by LPS and provides evidence of the possible beneficial health advantages of this native Taiwan fruit. On the basis of the results from the antioxidant experiments, we found that *P. emblica* fruit extracts showed excellent antioxidative activity, that immune cells could be regulated via *P. emblica* substances, and that, at low concentration, the fruit extract powder increased RAW 264.7 cell proliferations. LPS stimulation in RAW 264.7 cells enhanced the immunological activity on the accumulation of intracellular ROS and upregulations of inflammatory related genes (*NF-κB, iNOS,* and *COX-2*). *P. emblica* samples reduced the cellular ROS productions in a dose dependent manner from 0.125 to 2 mg/mL and decreased the above genes and proteins. *P. emblica* samples showed good antioxidant activities and anti-inflammation properties to be useful as a functional food additive.

## Figures and Tables

**Table 1:** Primers used for quantitative reverse transcription polymerase chain reaction for the analysis of inflammatory gene expressions.

***NF-κB***
Forward: 5′-TATGTGTGTGAAGGCCCATCA-3′
Reverse: 5′-ACCAACTGAACGATAACCTTTGC-3′
***iNOS***
Forward: 5′-CGAGACGGATAGGCAGAGATTG-3′
Reverse: 5′-CTCTTCAAGCACCTCCAGGAA-3′
***COX-2***
Forward: 5′-CCAGCACTTCACCCATCAGTTT-3′
Reverse: 5′-TCTGTCCAGAGTTTCACCATAAATG-3′

**Table 2:** The effect of antioxidative activity assays on *Phyllanthus emblica* at different concentrations.

Concentration (mg/mL)	DPPH Free Radical Scavenging Activity (%)	Reducing Power (OD_700_)	Metal Chelating Activity (%)
0	0 ± 0	0.105 ± 0.001	0 ± 0
0.5	3.92 ± 0.07	0.151 ± 0.001	5.66 ± 0.20
1	7.04 ± 0.10	0.180 ± 0.002	7.72 ± 0.69
2	16.43 ± 0.25	0.205 ± 0.002	11.63 ± 0.66
5	42.43 ± 0.51	0.436 ± 0.006	15.06 ± 0.13
10	67.03 ± 0.07	0.796 ± 0.023	16.12 ± 0.25
50	88.71 ± 0.30	2.311 ± 0.054	16.92 ± 0.11
Vitamin C ^a^	89.97 ± 0.17	-	-
BHA ^b^	-	0.604 ± 0.002	-
EDTA ^c^	-	-	94.43 ± 0.21

^a^ Vitamin C is the positive control of DPPH radical scavenging capacity assay with the concentration of 100 μM; ^b^ BHA is the positive control of reducing power assay with the concentration of 100 μM; ^c^ EDTA is the positive control of metal chelating activity assay with the concentration of 100 μM.
